# Oxyfunctionalization of pyridine derivatives using whole cells of *Burkholderia* sp. MAK1

**DOI:** 10.1038/srep39129

**Published:** 2016-12-16

**Authors:** Jonita Stankevičiūtė, Justas Vaitekūnas, Vytautas Petkevičius, Renata Gasparavičiūtė, Daiva Tauraitė, Rolandas Meškys

**Affiliations:** 1 Department of Molecular Microbiology and Biotechnology, Institute of Biochemistry, the Life Sciences Centre, Vilnius University, Sauletekio al. 7, LT-10257 Vilnius, Lithuania

## Abstract

Pyridinols and pyridinamines are important intermediates with many applications in chemical industry. The pyridine derivatives are in great demand as synthons for pharmaceutical products. Moreover, pyridines are used either as biologically active substances or as building blocks for polymers with unique physical properties. Application of enzymes or whole cells is an attractive strategy for preparation of hydroxylated pyridines since the methods for chemical synthesis of pyridinols, particularly aminopyridinols, are usually limited or inefficient. *Burkholderia* sp. MAK1 (DSM102049), capable of using pyridin-2-ol as the sole carbon and energy source, was isolated from soil. Whole cells of *Burkholderia* sp. MAK1 were confirmed to possess a good ability to convert different pyridin-2-amines and pyridin-2-ones into their 5-hydroxy derivatives. Moreover, several methylpyridines as well as methylated pyrazines were converted to appropriate *N*-oxides. In conclusion, regioselective oxyfunctionalization of pyridine derivatives using whole cells of *Burkholderia* sp. MAK1 is a promising method for the preparation of various pyridin-5-ols and pyridin-*N*-oxides.

The pyridine ring is found in various man-made compounds, such as dyes, industrial solvents, herbicides, pesticides as well as in many natural metabolites. Among the *N*-heterocyclic rings, pyridin-2-ol has considerable chemical and pharmacological importance. Due to a peptidomimetic functionality of 1H-pyridin-2-one tautomer, it plays an essential role as a scaffold in drug design^1^,^2^. Pharmacophores containing 1H-pyridin-2-ones are found in various therapeutic agents, including reverse transcriptase inhibitors^3^, antibiotics and antifungals^4^,^5^,^6^, anti-allergic drugs^7^ and analgesics^8^. Moreover, 1H-pyridin-2-ones are promising compounds for the preparation of modified nucleotides and oligonucleotides^9^,^10^,^11^.

A novel class of phenolic antioxidants, 6-aminopyridin-3-ols, are more effective than many other phenolic-class compounds reported to date^12^. Pyridin-2-amines serve as a starting material for production of fused heterocycles, including imidazo-derivatives that possess significant biological activities similar to those of antiviral and immunosuppressive agents^13^,^14^,^15^. Moreover, 3-amino-imidazo[1,2-a]pyridines were identified as a novel class of *Mycobacterium tuberculosis* glutamine synthetase inhibitors^16^, and 6-aminopyridin-3-ol was applied for the synthesis of new antibiotics^17^.

Preparation of some 5-hydroxy-2-pyridones is achievable by both organo-chemical and biocatalytic approaches^18^, whereas no satisfactory synthetic methods leading to 6-aminopyridin-3-ols have been described thus far. In addition, only a few synthetic methods to aminopyridinol structures may be found in literature to date^12^,^19^,^20^. Recently, a chemical synthesis of pyridine-3,5-diol derivatives from renewable carbohydrates has been demonstrated^21^.

Oxyfunctionalization of chemical compounds by using enzymes or whole cells is an attractive strategy to obtain the desired products^22^,^23^,^24^. The degradation of *N*-heterocyclic compounds, especially with regard to the production of metabolic intermediates, has received considerable attention in biotechnology as the starting process for the synthesis of fine and commodity chemicals, e.g., pyridine-2,5-diol (an intermediate for production of 5-aminolevulinic acid), 6-hydroxynicotinic acid and other pyridine derivatives^25^,^26^,^27^,^28^. Recently, the hydroxylation of the pyridine ring has been achieved using tetramethylpyrazine-degrading bacteria^27^. It has been also shown that pyridine *N*-oxides can be prepared using microbial cells and enzymes; however, this has been accomplished using *Methylococcus capsulatus* monooxygenase^29^, *Agrocybe aegerita* peroxygenase^30^, and *Verticillium* sp. GF39 cells^31^ only.

Several bacteria belonging to the genera *Arthrobacter, Achromobacter, Rhodococcus*, and *Nocardia* are able to grow on pyridin-2-ol^32^,^33^,^34^,^35^. The first common steps in the microbial metabolism of pyridin-2-ol involve the hydroxylation of the ring yielding di- or trihydroxypyridine intermediates^32^,^35^,^36^ that are promising synthons for the preparation of substituted pyridines.

In this study, the oxyfunctionalization of the pyridine ring by whole bacterial cells was investigated. The pyridin-2-ol-degrading *Burkholderia* sp. MAK1 was found to be an efficient biocatalyst for the hydroxylation of various pyridin-2-ols and pyridin-2-amines. Moreover, *Burkholderia* sp. MAK1 was capable of oxidising several *N*-heterocyclic ring systems to corresponding *N*-oxides.

## Results and Discussion

### Isolation of pyridin-2-ol-degrading bacteria

The gram-negative bacterial isolate MAK1, capable of using pyridin-2-ol as a sole carbon and energy source, was isolated from soil. The 16S rRNA gene sequence of MAK1 showed similarity to that of bacteria belonging to *Burkholderia sordidicola*. Based on the results of 16S rRNA gene sequence analysis (Supplementary information Fig. S-I) and biochemical characterization (Supplementary information Table S-1) the strain MAK1 was identified as *Burkholderia* sp. MAK1.

In bacteria, pyridin-2-ol may be catabolized by two different pathways. The first pathway proceeds via formation of pyridine-2,3,6-triol, which spontaneously oxidises and dimerises to a blue pigment, 4,5,4′,5′-tetrahydroxy-3,3′-diazadiphenoquinone-(2,2′)^32^,^35^,^37^. The other known catabolic pathway proceeds via formation of pyridine-2,5-diol, maleamic acid, maleic acid, and fumaric acid^33^.

In the case of *Burkholderia* sp. MAK1 described here, pyridin-2-ol was catabolized without the formation of a blue pigment. Assuming that pyridine-2,5-diol is an intermediate in pyridin-2-ol catabolic pathway, the activity of pyridine-2,5-diol 5,6-dioxygenase detected in the pyridin-2-ol-induced cells of *Burkholderia* sp. MAK1 suggested that this strain possesses an inducible pyridin-2-ol 5-monooxygenase.

### Selection of pyridine derivatives as substrates for hydroxylation with *Burkholderia* sp

As we found out that *Burkholderia* sp. MAK1 consumes pyridine-2-ol via pyridine-2,5-diol by supposedly pyridine-2-ol inducible pyridin-2-ol 5-monooxygenase we wanted to test whether *Burkholderia* sp. MAK1 is capable of hydroxylating other pyridine derivatives. In this study, more than 100 of pyridine, pyrimidine, and pyrazine derivatives were screened for the hydroxylation using *Burkholderia* sp. MAK1 as a whole-cell biocatalyst (Supplementary information Table S-2). The pyridin-2-ol-induced *Burkholderia* sp. MAK1 cells were incubated with a potential substrate as described in the Methods section. The progress of the reaction was followed by HPLC-MS. The efficiency of conversion of several compounds by whole cells of *Burkholderia* sp. MAK1 is presented as Supplementary information Table S-3.

It is worth mentioning that induction of *Burkholderia* sp. MAK1 hydroxylation activity was observed only in the presence of pyridin-2-ol. Several other tested compounds (pyridine, pyridine-2,5-diol, pyridin-2-amine) were not able to trigger the induction. Also no hydroxylation occurred when cells were cultivated with other sole carbon source (glucose or succinate) instead of pyridin-2-ol.

### Optimization of cultivation and reaction conditions

**Figure 1:**
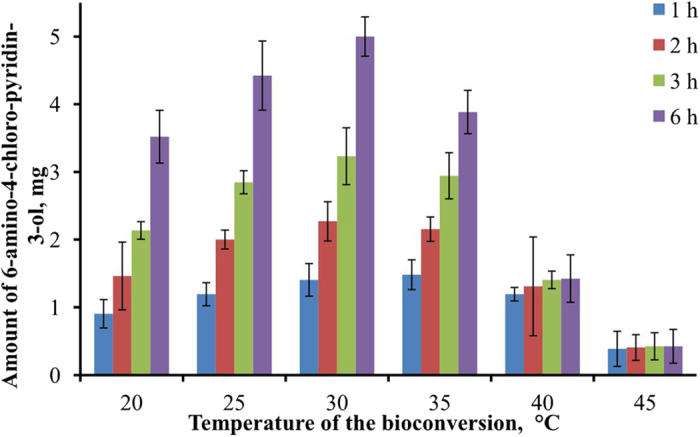
The dependence of the rate of 6-amino-4-chloro-pyridin-3-ol biosynthesis on temperature. 2-hydroxypyridine-induced *Burkholderia* sp. MAK1 cells were used as biocatalyst. The values represent the average of three independent experiments ± standard deviation.

*Burkholderia* sp. MAK1 grew poorly in rich nutrient medium, but the growth was observed in mineral medium (EFA or Koser) with pyridin-2-ol as a sole carbon source. The growth reached its peak after 40 h of incubation in EFA medium (OD_600_ = 0.4). The optimal temperature for cultivation of *Burkholderia* sp. MAK1 appeared to be 30 °C. At higher tested temperature (37 °C), *Burkholderia* sp. MAK1 cells were not able to grow. Although bacterial growth was observed at 25 °C it was rather slow compared to 30 °C. The effect of temperature on *Burkholderia* sp. MAK1-mediated synthesis of hydroxylated pyridine derivatives was also investigated (Fig. 1). For this experiment 4-chloropyridin-2-amine was selected due to its great conversion percentage and definite product (Table 1). During the first hour of the experiment, the bioconversion of 4-chloropyridin-2-amine was most rapid at 30 °C and 35 °C with 6-amino-4-chloro-pyridin-3-ol production rate of 7 mg (g biomass)-1 h-1 and 7.4 mg (g biomass)-1 h-1, respectively. Higher temperatures (40–45 °C) were found to be unfavorable for the synthesis, probably because of the inactivation of the biocatalyst. The conversion reached near completion (~97%) after six hours at 30 °C.

### Biotransformation of various pyridin-2-ols by *Burkholderia* sp. MAK1 cells

The study of *N*-alkylpyridine transformation revealed that 1-methyl-, 1-ethyl- and 1-propylpyridin-2-ol were transformed to the final dihydroxy products by *Burkholderia* sp. MAK1 cells. In the chromatogram of 1-ethylpyridin-2-ol bioconversion, two dominant peaks A and B were detected (Supplementary information Fig. S-II) corresponding to the newly formed compound and the residual substrate, respectively. The absorption maximum of the product, compared with that of the substrate, shifted to longer wavelengths (~30 nm), which is characteristic of compounds with additional hydroxy group. Also, the mass of the molecular ion of the product was 16 Da higher than that of the parent compound, supporting the hydroxylation of 1-ethylpyridin-2-ol. Similar results were obtained with 1-methyl- and 1-propylpyridin-2-ol. In all cases, the formation of a single product was observed indicating the position-specific hydroxylation. Moreover, the apparent equivalence with pyridin-2-ol transformation suggested that 1-alkylpyridin-2-ols were hydroxylated at the 5-position. Of all the compounds tested, only 1-butylpyridin-2-ol remained unchanged, which is most likely due to its bulkiness. In summary, pyridin-2-ols containing small 1-alkyl substituent are hydroxylated regioselectively, but further pyridine ring opening reaction does not occur. Thus, *Burkholderia* sp. MAK1 is capable of producing 1-alkylpyridine-2,5-diols.

**Figure 2:**
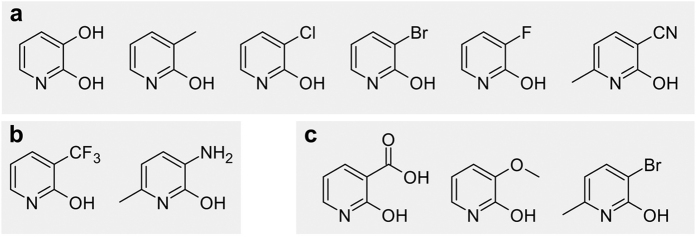
The 2-hydroxypyridines substituted at the third position, which were used as potential *Burkholderia* sp. MAK1 substrates. (**a**) Substrate consumption occurs, no products detected, (**b**) substrate consumed, product detected, (**c**) no reaction observed. The 2-hydroxypyridine induced cells were suspended in 10 mM potassium phosphate buffer, pH 7.2, supplemented with 15 mM glucose and 0.25 mM of corresponding substrate. Reactions were carried out at 30 °C. The progress of each reaction was observed by HPLC-MS.

Another group of potential *Burkholderia* sp. MAK1 substrates comprised pyridin-2-ols substituted at position 3 (Fig. 2). HPLC-MS analysis revealed that compounds containing hydroxyl, methyl, bromo, chloro, or fluoro functional groups were completely catabolized by *Burkholderia* sp. MAK1 cells since no significant peaks corresponding to any hydroxylated products were detected. The latter suggests that the hydroxylated metabolites were likely further metabolized to aliphatic products. However, 3-(trifluoromethyl)pyridin-2-ol was slowly converted into a detectable new compound whose molecular mass was 16 Da higher than that of the substrate. *Burkholderia* sp. MAK1 cells were not able to hydroxylate pyridin-2-ols containing carboxyl or methoxy groups at position 3.

Pyridin-2-ols carrying substituents at positions 3 and 6 were also examined. The pyridin-2-ol-induced cells were able to metabolize 2-hydroxy-6-methyl-pyridine-3-carbonitrile: substrate concentration decreased over time, and no new products were detectable by HPLC-MS. After incubation of *Burkholderia* sp. MAK1 with 3-amino-6-methyl-pyridin-2-ol, a new compound with a molecular mass of 278 Da accumulated in the reaction mixture. Since the molecular mass of the expected 3-amino-6-methyl-pyridin-2-ol hydroxylation product is 140 Da, it is likely that the oxidation of the substrate is followed by the spontaneous dimerization. When *Burkholderia* sp. MAK1 cells were incubated with 3-bromo-6-methyl-pyridin-2-ol, neither hydroxylation, nor any other transformation occurred suggesting that 3-bromo functional group disrupted the proper orientation of the substrate.

**Figure 3:**
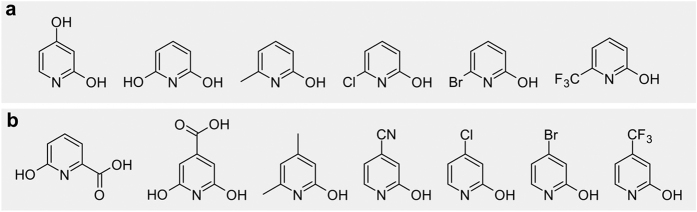
2-Hydroxypyridine derivatives substituted at the fourth, the sixth or both the fourth and the sixth positions, which were used as potential substrates for regioselective oxidation by *Burkholderia* sp. MAK1. (**a**) Substrate consumption occurs; (**b**) no reaction observed. The 2-hydroxypyridine induced cells were suspended in 10 mM potassium phosphate buffer, pH 7.2, supplemented with 15 mM glucose and 0.25 mM of corresponding substrate. Reactions were carried out at 30 °C. The progress of each reaction was observed by HPLC-MS.

Pyridin-2-ols substituted at positions 4 and/or 6 were also used as substrates in this study (Fig. 3). Pyridine-2,4-diol was completely oxidized by *Burkholderia* sp. MAK1 cells after 20 hours of incubation. However, the intermediate product accumulating in the reaction mixture was detected by HPLC-MS and its absorption spectra as well as molecular mass ([M + H]^+^ = 128.05, [M + H_2_O + H]^+^ = 146.10, [2M+ H]^+^ = 255.05) were consistent with those of hydroxylated pyridine-2,4-diol (Supplementary information Fig. S-III). Using 4-cyano, 4-chloro, 4-bromo, or 4-trifluomethyl substituted pyridin-2-ols, hydroxylation of the pyridine ring did not occur suggesting that the nature of a substituent at position 4 is important for the hydroxylation process.

Pyridine-2,6-diol was transformed by *Burkholderia* sp. MAK1 to a blue pigment. Previously, Holmes with colleagues described dimerization of pyridine-2,3,6-triol, which led to the formation of a blue pigment^38^. Following this observation, the hydroxylation of the symmetric pyridine-2,6-diol by *Burkholderia* sp. MAK1 cells likely occurred at position 3 of the pyridine ring and the resulting pyridine-2,3,6-triol spontaneously dimerized to a blue compound. Moreover, if the sixth position of pyridin-2-ol was occupied by a small and uncharged functional group, the pyridine ring cleavage probably followed the hydroxylation event.

Summarizing experiments with substituted pyridin-2-ols we can make the statement that most of the substrates were consumed without detectable products. Although we were unable to provide any data about structures of the detectible product there were strong evidences suggesting regioselective hydroxylation at 5-position (Table 2).

### Screening of pyridin-2-amines as potential substrates for regioselective hydroxylation by *Burkholderia* sp. MAK1 cells

The ability of *Burkholderia* sp. MAK1 to transform various pyridin-2-ols encouraged us to study pyridin-2-amines as another group of potential substrates. During the initial experiments, the cells were incubated with pyridin-2-amine for 20 hours. HPLC-MS analysis revealed that pyridin-2-amine was completely consumed, and the new peak in the chromatogram belonged to the expected product. The molecular mass of the product, which was 16 Da higher than that of pyridin-2-amine, confirmed the notion that hydroxylation of the substrate occurred. The UV-Vis spectrum of the product was compared with spectra of commercially available reference standards (pyridin-2-amine hydroxylated at position 3, 4, or 6), yet none of these spectra matched that of the product (Supplementary information Fig. S-IV). From this we presume that in the case of *Burkholderia* sp. MAK1, pyridin-2-amine undergoes hydroxylation at position 5.

Next, pyrazin-2-amine, a homolog of pyridin-2-amine containing two nitrogen atoms in the aromatic ring, was chosen as a substrate for the bioconversion. HPLC-MS analysis showed that the molecular mass of the biotransformation product was 16 Da higher than that of pyrazin-2-amine, suggesting that *Burkholderia* sp. MAK1 cells are also capable of pyrazin-2-amine hydroxylation.

**Figure 4:**
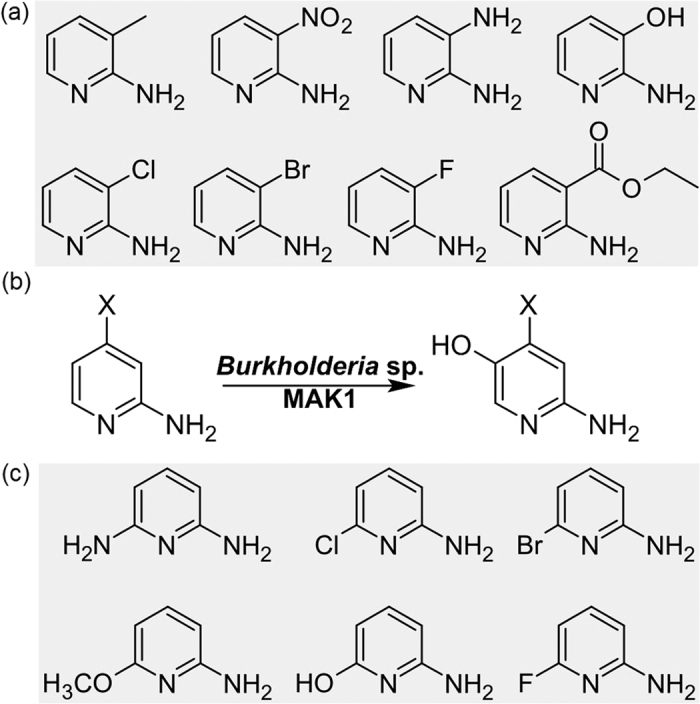
The 2-aminopyridines, which were tested as potential substrates for conversion by 2-hydroxypyridine-induced whole cells of *Burkholderia* sp. MAK1. (**a**) The 2-aminopyridine derivatives substituted at the third position; (**b**) the regioselective hydroxylation of 2-aminopyridines substituted at the fourth position (X = CH_3_, Cl, F); (**c**) the 2-aminopyridines substituted at the sixth position.

Pyridin-2-amines with methyl, nitro, chloro, bromo, or fluoro substituent at position 3 (Fig. 4a) were all transformed by *Burkholderia* sp. MAK1. Moreover, the pyridin-2-ol-induced cells were also capable of hydroxylating ethyl-2-aminopyridine-3-carboxylate, a compound with a bulky functional group at the 3-position. The conversion product of 3-chloropyridin-2-amine was purified as described in the Materials and Methods section, and its structure was analysed by ^1^H NMR, ^13^C NMR, and HPLC-MS analyses. The molecular mass of the product (145 Da) corresponded to that of 6-amino-5-chloro-pyridin-3-ol. The compound showed four peaks in the 1H NMR spectrum (DMSO-d_6_, ppm): δ = 5.51 (s, 2 H, NH_2_), 7.11 (d, J = 2.6 Hz, 1H, CH), 7.56 (d, J = 2.6 Hz, 1H, CH), 9.24 (brs, 1H, OH), and five peaks in the 13C NMR spectrum (DMSO-d_6_, ppm): δ = 113.58, 125.21, 133.73, 146.21, 149.46), identifying the product as 6-amino-5-chloro-pyridin-3-ol. The production yield of 6-amino-5-chloro-pyridin-3-ol was 34%.

Both pyridine-2,3-diamine and 2-aminopyridin-3-ol were transformed into colored compounds, with a molecular mass of 213 Da (yellow-brown) and 214 Da (yellow-green), respectively. The retention time, UV-Vis spectra, and ionisation profile of the oxidation product of 2-aminopyridin-3-ol matched those of the analytical standard (2-amino-3*H*-dipyrido[3,2-b:2′,3′-e][1,4]oxazine-3-one) suggesting that *Burkholderia* sp. MAK1 catalyzes the oxidative dimerization of 2-aminopyridin-3-ol. Also, although another analytical standard, pyridine-2,3-diamine derivative, is commercially unavailable, our results indicate, that MAK1 catalyzes dimerization of pyridine-2,3-diamine as well. These dimers are potential anticancer and antimicrobial drugs^39^.

Next, the ability of *Burkholderia* sp. MAK1 cells to transform pyridin-2-amines substituted at position 4 was investigated. Compounds with methyl, chloro, bromo, or fluoro substituents were hydroxylated. In all cases, the molecular mass of reaction products, as estimated by HPLC-MS, was 16 Da higher than that of parent compounds indicating that oxidation of substrates had occurred.

In the case of 4-methyl-pyridin-2-amine, 4-chloro-pyridin-2-amine, and 4-fluoro-pyridin-2-amine, the biotransformation catalyzed by the pyridin-2-ol-induced *Burkholderia* sp. MAK1 cells resulted in the formation of a single product. The products of all three reactions were purified by a reverse phase chromatography (C18 cartridges, water/methanol mixture, 10:0 → 10:5), and their structures were analysed by ^1^H NMR and ^13^C NMR. 6-Amino-4-methyl-pyridin-3-ol (^1^H NMR (DMSO-d_6_, ppm): δ = 2.18 (s, 3H, CH_3_), 6.41 (dd, J = 6.6, 2.3 Hz, 1H, CH), 6.61 (d, J = 2.3 Hz, 1H, CH), 6.70 (s, 2H, NH_2_), 7.87 (d, J = 6.6 Hz, 1H, CH), ^13^C NMR (DMSO-d_6_, ppm): δ = 20.52, 109.39, 113.72, 136.73, 138.00, 150.61), 6-amino-4-chloro-pyridin-3-ol (^1^H NMR (DMSO-d_6_, ppm): δ = 6.66 (dd, J = 7.0, 2.9 Hz, 1H, CH), 6.84–6.83 (m, 1H, CH), 7.0 (s, 2H, NH_2_), 8.04 (d, J = 7.0 Hz, 1H, CH), ^13^C NMR (DMSO-d_6_, ppm): δ = 108.17, 112.44, 131.29, 138.34, 151.70) and 6-amino-4-fluoro-pyridin-3-ol (^1^H NMR (DMSO-d_6_, ppm): δ = 6.54 (td, J = 7.3, 3.4 Hz, 1H, CH), 6.62 (dd, J = 9.1, 3.2 Hz, 1H, CH), 7.11 (s, 2H, NH_2_), 8.07 (dd, J = 7.2, 6.0 Hz, 1H, CH), ^13^C NMR (DMSO-d_6_, ppm): δ = 95.45, 100.74, 139.03, 158.91, 161.39) were formed by whole cells of *Burkholderia* sp. MAK1 with the yield of 34%, 50% and 68%, respectively (Fig. 4b). In addition, 4-chloropyrimidin-2-amine, pyrimidine-2,4-diamine, and 2-aminopyrimidin-4-ol were also hydroxylated by the pyridin-2-ol-induced *Burkholderia* sp.MAK1 cells. According to the ^1^H NMR and ^13^C NMR analyses, the purified product of 2-aminopyrimidin-4-ol conversion was 2-aminopyrimidine-4,5-diol, and the conversion yield was 18%. To our knowledge, biocatalytical production of 6-amino-4-methyl-pyridin-3-ol has never been described previously. Moreover, there is no available information concerning the synthesis of 6-amino-4-chloro-pyridin-3-ol or 6-amino-4-fluoro-pyridin-3-ol. By analogy to aminophenols, the new compounds described in this study have great potential as materials for the production of dyes, drugs, pesticides, and etc.^40^.

The compounds substituted at position 6 (Fig. 4c) were also transformed by *Burkholderia* sp. MAK1. HPLC-MS analysis showed that pyridine-2,6-diamine was consumed; however, no new compounds were detected. Nevertheless, in the case of pyridine-2,6-diamine, the reaction mixture turned brown suggesting that after oxidation, further transformations (e. g. polymerisation) occurred. The compounds with 6-chloro or 6-bromo substituents were converted to the corresponding hydroxylated products. The product of oxidation of 6-chloropyridin-2-amine, 6-amino-2-chloropyridin-3-ol, was purified and identified by ^1^H NMR (DMSO-d_6_, ppm): δ = 5.90 (s, 2H, NH_2_), 6.38 (d, J = 7.8 Hz, 1H, CH), 6.84 (d, J = 7.8 Hz, 1H, CH), 9.79 (brs 1H, OH). While 6-fluoropyridin-2-amine conversion was very slow, the transformation of 6-methoxypyridin-2-amine did not occur at all. The conversion of 6-aminopyridin-2-ol led to several compounds suggesting that the substrate is hydroxylated at position 3 and/or 5, so that a mixture of several products in varying proportions results.

Unlike the aforementioned pyridin-2-ols, the products of hydroxylation of 6-substituted pyridin-2-amines were not metabolised further suggesting that *Burkholderia* sp. MAK1 may be applied for the regioselective synthesis of 6-substituted 2-aminopyridinols (Table 1).

### Oxyfunctionalization of pyridine, pyrazine and their derivatives using whole-cell biocatalyst

The study on pyridin-2-amine and pyridin-2-ol bioconversion by *Burkholderia* sp. MAK1 cells showed that the pyridin-2-ol-inducible pyridin-2-ol 5-monooxygenase has broad substrate specificity and strict regiospecificity since it catalyzes hydroxylation at position 5 on the aromatic ring. With very few exceptions, microbial hydroxylation of pyridine-2-amines has been scarcely studied. One such exception is the study on the biotransformation of 4-methyl-3-nitro-pyridin-2-amine using whole-cells of fungus *Cunninghamella elegans* ATCC 26269. During this biotransformation, a mixture of three products, 6-amino-4-methyl-5-nitropyridin-3-ol, 2-amino-4-hydroxymethyl-3-nitropyridine, and 2-amino-4-methyl-3-nitropyridine-1-oxide was obtained suggesting that both aromatic and aliphatic positions as well as the heterocyclic nitrogen atom undergo oxidation^41^. In the case of *Burkholderia* sp. MAK1 cells, oxidation of the heterocyclic nitrogen atom was not observed when pyridin-2-ols were used as substrates. To determine if these bacteria were capable of producing *N*-oxides, various pyridine and pyrazine compounds without amino or hydroxy group at position 2 were tested as substrates for pyridin-2-ol-induced *Burkholderia* sp. MAK1 cells. HPLC-MS analysis showed that pyridine was transformed into a single product whose molecular mass was 16 Da higher than that of the parent compound. The UV spectrum of the product was very similar to that of pyridine yet did not match with the spectra of 2-, 3-, or 4-hydroxy-substituted pyridines at position suggesting that the product of pyridine biotransformation is pyridine-1-oxide (pyridine-*N*-oxide). The retention time, UV spectrum and ionisation profile of the bioconversion product matched those of analytical standard, pyridine-*N*-oxide, suggesting that *Burkholderia* sp. MAK1 catalyzes pyridine oxidation at position 1. Induction of cells with pyridin-2-ol was necessary for the oxidation of pyridine as well as for pyridin-2-ol and pyridin-2-amine transformation indicating that the same enzyme of *Burkholderia* sp. MAK1 is responsible for all these biotransformations.

**Figure 5:**
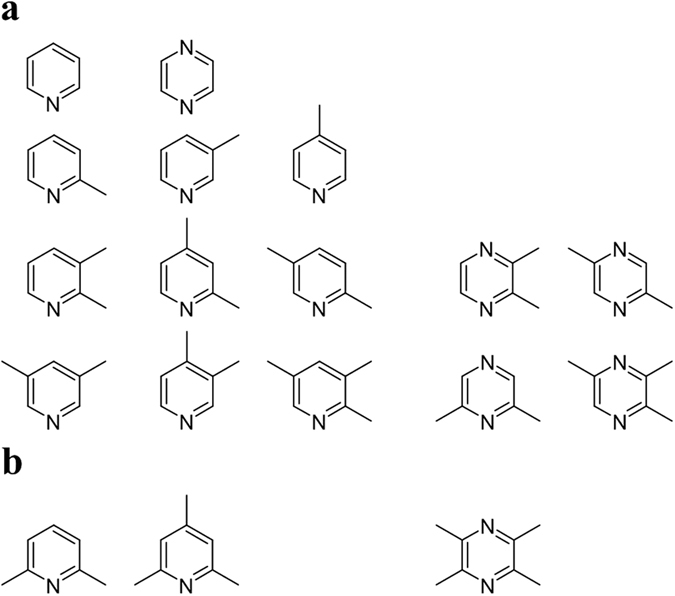
Pyridine, pyrazine and their methylated derivatives as substrates for the 2-hydroxypyridine-induced *Burkholderia* sp. MAK1 cells. (**a**) Substrate consumption occurs; (**b**) no reaction observed.

A group of pyridines and pyrazines containing a methyl group attached to the aromatic ring at different positions (Fig. 5) was studied as potential substrates for *Burkholderia* sp. MAK1. The test revealed that the whole cells of *Burkholderia* sp. MAK1 catalyzed the transformation of 2-methyl-, 3-methyl-, and 4-methylpyridine into corresponding *N*-oxides whose structures were confirmed by HPLC-MS using analytical standards (Table 3). *Burkholderia* sp. MAK1 was also capable of transforming di- and trimethyl pyridines, except those in which both positions adjacent to nitrogen were occupied.

Based on HPLC-MS analysis, the biotransformation of pyrazine resulted in the formation of two products with molecular masses that were 16 Da and 32 Da higher than that of the parent compound. ^1^H and ^13^C NMR analysis allowed identification of these products as pyrazine-1-oxide (^1^H NMR (DMSO-d_6_, ppm): δ = 8.34–8.36 (m, 2H, CH), 8.54–8.57 (m, 2H, CH); ^13^C NMR (DMSO-d_6_, ppm): δ = 134.85, 148.94) and pyrazine-1,4-dioxide (^1^H NMR (DMSO-d_6_, ppm): δ = 8.28 (s, 4H, CH); ^13^C NMR (DMSO-d_6_, ppm): δ = 137.21).

Our research revealed that *Burkholderia* sp. MAK1 has also the ability to oxidize various methylpyrazines. For the oxidation of methylated pyrazines the single free position adjacent to either one of nitrogen atoms was a sufficient condition, e. g. the cells could oxidize 2,3,5-trimethylpyrazine, but not 2,3,5,6-tetramethylpyrazine.

To date, only a few reports regarding the microbial *N*-hydroxylation of pyridines have been published. The formation of pyridine *N*-oxides has been observed in fungi *Cunninghamella elegans* ATCC 26269^41^, *Verticillium* sp. GF39^31^, and other fungi^42^ as well as in bacteria *Methylococcus capsulatus*^29^ and *Diaphorobacter* sp. J5-51^43^. Also, the purified aromatic peroxygenase from *fungus Agrocybe aegerita* has been found to be active towards pyridine and its derivatives^30^. In this context, the results of this study not only broaden our understanding of microbial transformation but also provide a versatile tool that can be used in a regioselective oxyfunctionalization of various pyridine derivatives.

## Conclusions

**Figure 6:**
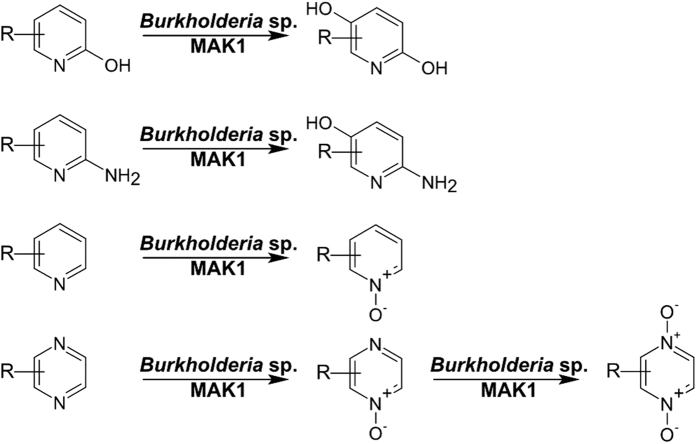
The general scheme of oxyfunctionalization of pyridine and pyrazine derivatives using whole cells of *Burkholderia* sp. MAK1.

In summary, whole cells of *Burkholderia* sp. MAK1 have high activity towards pyridin-2-amines and pyridin-2-ols, and are applicable for the synthesis of pyridin-5-ols from the corresponding substrates. Moreover, unsubstituted pyridine and pyrazine as well as their methylated derivatives can be converted into the corresponding *N*-oxides using pyridin-2-ol-induced *Burkholderia* sp. MAK1 (Fig. 6). The approach presented here offers a promising alternative to chemical synthesis of hydroxylated pyridines.

## Methods

### Chemicals

Pyridin-2-amine, 2-chloropyridine, pyridine-2-carboxylic acid, pyridine-*N*-oxide, pyridin-3-ol, 2-aminopyridin-3-ol, 3-nitropyridin-2-amine, 2-hydroxy-6-oxo-1H-pyridine-4-carboxylic acid, pyrazine, pyridine-4-carboxylic acid and pyridine-2,3-dicarboxylic acid were purchased from Merck (Darmstadt, Germany). Pyridin-2-ol, pyrimidin-2-ol, 2-methylpyridine, 3-methylpyridine, 4-methylpyridine, 2-pyridylmethanol, pyridine-2-thiol, 1-methylpyridin-2-one, 3-bromopyridin-2-ol, 3-methylpyridin-2-ol, 3-methoxypyridin-2-ol, pyridine-2,3-diol, pyridine-2,4-diol, pyridine-2,6-diol, 3-methylpyridin-2-amine, 3-methylpyridine-2-carbonitrile, 2,3-dimethylpyridine, 2,4-dimethylpyridine, 2,5-dimethylpyridine, 2,6-dimethylpyridine, 3,4-dimethylpyridine, 3,5-dimethylpyridine, 2,3,5-trimethylpyridine, pyridine-2,3-diamine, pyridine-2,6-diamine, 4-methyl-3-nitro-pyridin-2-ol, 4-methylpyridin-2-amine, 4,6-dimethylpyridin-2-amine, 3-methylpyridin-6-amine, pyridine, pyran-2-one, 2-hydroxy-6-methyl-pyridine-3-carbonitrile, 1-pyrazin-2-ylethanone, 2,3-dimethylpyrazine, 2,5-dimethylpyrazine, 2,6-dimethylpyrazine, 2,3,5-trimethylpyrazine, pyrazin-2-amine, tetramethylpyrazine, 2-(2-pyridyl)ethanol, 2-aminopyridine-3-carbonitrile, 3-methylpyridazine, 2-methylpyridin-3-ol, 4-methylpyridine-2-carbonitrile, pyridine-2,6-dicarbonitrile, 6,7-dihydro-5H-cyclopenta[b]pyridine, 5-methyl-6,7-dihydro-5H-cyclopenta[b]pyrazine, pyrimidine-2,4-diamine, 2-methylpyridin-5-ol, 2-aminopyridin-6-ol, 4-methyl-3-nitro-pyridin-2-amine were obtained from Sigma-Aldrich (St. Louis, MO). Pyridin-2-ol *N*-oxide, 2-hydroxypyridine-3-carboxylic acid, 6-hydroxypyridine-2-carboxylic acid, 3-hydroxypyridine-2-carboxylic acid, 6-methylpyridin-2-ol, 2-methylpyridine *N*-oxide, 2-ethylpyridine, 4-ethylpyridine, pyridine-3-carboxamide, 4-[(4-nitrophenyl)methyl]pyridine, pyridine-3-carbonitrile, 2,4,6-trimethylpyridine and pyridin-4-ol were purchased from Fluka (Buchs, Switzerland). 5-hydroxypyridine-2-carboxylic acid, 3-fluoropyridin-2-ol, 3-chloropyridin-2-ol, 6-chloropyridin-2-ol, 6-bromopyridin-2-ol, 6-methylpyridin-2-ol, 4-chloropyridin-2-ol, 2-oxo-1H-pyridine-4-carbonitrile, 4,6-dimethylpyridin-2-ol, 3-amino-6-methyl-pyridin-2-ol, 3-(trifluoromethyl)pyridin-2-ol, 4-(trifluoromethyl)pyridin-2-ol, 6-(trifluoromethyl)pyridin-2-ol, 6-(trifluoromethyl)pyridin-2-amine, 3-chloropyridin-2-amine, 4-chloropyridin-2-amine, 6-chloropyridin-2-amine, 3-bromopyridin-2-amine, 4-bromopyridin-2-amine, 6-bromopyridin-2-amine, 3,6-dibromopyridin-2-amine, 3-fluoropyridin-2-amine, 4-fluoropyridin-2-amine, 6-fluoropyridin-2-amine, 2-aminopyridin-4-ol, 3-methoxypyridin-2-amine, 6-methoxypyridin-2-amine, ethyl 2-aminopyridine-3-carboxylate, 2-aminopyrimidin-4-ol, 4-chloropyrimidin-2-amine, 4-bromopyridin-2-ol and 3-bromo-6-methylpyridin-2-ol were the products of Combi Blocks Inc (San Diego, USA). All reagents used in this study were of analytical grade. Nutrient agar (NA) medium and brain heart infusion (BHI) medium were obtained from Oxoid (Hampshire, UK). The 2-amino-3H-dipyrido[3,2-b:2’,3’-e][1,4]oxazine-3-one prepared by oxidation-dimerization of 2-amino-3-hydroxypyridine as described for 2-aminophenol^44^ was a gift from Dr. J. Šarlauskas. The alkylated pyridones were synthesized according to the published procedure^45^.

1-Ethylpyridin-2(1*H*)-one. Yield 170 mg (69%), colourless oil. MS (ESI^+^): *m/z* 124.15 [M + H]^+^. ^1^H NMR (CDCl_3_, ppm): δ = 1.37 (t, *J* = 7.2 Hz, 3H, CH_3_), 4.00 (q, *J* = 7.2 Hz, 2H, CH_2_), 6.21 (td, *J* = 6.7, 1.4 Hz, 1H, CH), 6.61 (ddd, *J* = 9.1, 1.4, 0.8 Hz, 1H, CH), 7.29–7.37 (m, 2H, CH); ^13^C NMR (CDCl_3_, ppm): δ = 14.69, 44.96, 106.47, 120.96, 137.01, 139.46, 162.51.

1-Propylpyridin-2(1*H*)-one. Yield 200 mg (73%), colourless oil. MS (ESI^+^): *m/z* 138.15 [M + H]^+^. ^1^H NMR (DMSO-d_6_, ppm): δ = 0.95 (t, *J* = 7.4 Hz, 3H, CH_3_), 1.56–1.69 (m, 2H, CH_2_), 3.75 (t, *J* = 7.4 Hz, 2H, CH_2_), 6.11 (td, *J* = 6.7, 1.4 Hz, 1H, CH), 6.42 (ddd, *J* = 9.1, 1.3, 0.6 Hz, 1H, CH), 7.35–7.46 (m, 1H, CH), 7.72 (ddd, *J* = 6.7, 2.1, 0.6 Hz, 1H, CH); ^13^C NMR (DMSO-d_6_, ppm): δ = 14.00, 29.23, 46.78, 105.55, 119.82, 138.93, 141.43, 162.15.

1-Butylpyridin-2(1*H*)-one. Yield 180 mg (60%), yellowish oil. MS (ESI^+^): *m/z* 152.20 [M + H]^+^. ^1^H NMR (DMSO-d_6_, ppm): δ = 0.90 (t, *J* = 7.4 Hz, 3H, CH_3_), 1.18–1.34 (m, 2H, CH_2_), 1.52–1.65 (m, 2H, CH_2_), 3.86 (t, *J* = 7.4 Hz, 2H, CH_2_), 6.19 (td, *J* = 6.7, 1.4 Hz, 1H, CH), 6.36 (ddd, *J* = 9.1, 1.4, 0.6 Hz, 1H, CH), 7.33–7.42 (m, 1H, CH), 7.66 (ddd, *J* = 6.7, 2.1, 0.6 Hz, 1H, CH); ^13^C NMR (DMSO-d_6_, ppm): δ = 14.05, 19.70, 31.25, 48.61, 105.50, 120.02, 139.53, 140.13, 161.82.

### Microbial cultures and cultivation conditions

EFA (g/l): K_2_HPO_4_ 10.0, KH_2_PO_4_ 4.0, yeast extract 0.5, (NH_4_)_2_SO_4_ 1.0, 2-hydroxypyridine 2.0, MgSO_4_ × 7H_2_O 0.2, salt solution 10 ml/l., pH 7.2; Salt solution (g/l): CaCl_2_ × 2H_2_O 2.0, MnSO_4_ × 4H_2_O 1.0, FeSO_4_ × 7H_2_O 0.5, all components were dissolved in 0.1N HCl and added in to EFA medium before cultivation; Koser mineral medium (g/l): NaCl 5.0, NH_4_H_2_PO_4_ 1.0, K_2_HPO_4_ 1.0, MgSO_4_ × 7H_2_O 0.4. The final pH was adjusted to 7.0^46^. Koser agar medium was prepared adding agar to Koser mineral medium (15 g/l). Nutrient agar medium (g/l): 28.0; BHI (g/l): 37. All media and solutions were autoclaved at 1 atm for 30 min.

Bacteria were cultivated in liquid media with aeration at 30 °C.

For substrate specificity and bioconversion experiments *Burkholderia* sp. MAK1 was grown at 30 °C for 20 hours in 1 l flasks containing 200 ml EFA medium. The cells were harvested by centrifugation and washed twice with 10 mM potassium phosphate buffer, pH 7.2.

### Isolation of 2-hydroxypyridine utilizing microorganisms

0.5 g of soil was suspended in 20 ml of Koser mineral medium. The aliquots (10–100 μl) were spread on Koser agar plates supplemented with 0.1% 2-hydroxypyridine and clotrimazole (20 μg/ml). Clotrimazole is known as cytochrome P450 inhibitor and was used to suppress growth of actinobacteria (e. g., *Rhodococcus, Streptomyces, Mycobacterium*) or fungi. After 3–5 days of aerobical cultivation at 30 °C the best growing colonies were selected for further work.

### DNA analysis

DNA was extracted according to Woo *et al*.^47^. 16S rRNA encoding genes were amplified using universal primers w001 and w002^48^. The PCR product was cloned into the pTZ57R/T plasmid (Thermo Fisher Scientific, Lithuania) and sequenced using M13/pUC (-46) forward 22-mer and reverse 24-mer primers. The 16S rRNA sequence of MAK1 was analyzed using BLAST tool and The Ribosomal Database Project in the NCBI database. A phylogenetic tree was constructed and displayed using the neighbor-joining method with MEGA6^49^. The *Burkholderia* sp. MAK1 16S rRNA gene sequence was deposited in GenBank under accession no. KU195413. *Burkholderia* sp. MAK1 was deposited to DSMZ German Culture Collection with accession no. DSM102049.

### Bioconversion of pyridines, pyrimidines and pyrazines using the cells of *Burkholderia* sp. MAK1

0.05 g of wet biomass of *Burkholderia* sp. MAK1 cells was resuspended in 1 ml of 10 mM potassium phosphate buffer, pH 7.2. The suspension was supplemented with 15 mM glucose and 0.25 mM of corresponding substrate and incubated at 30 °C. The process of the conversion was followed by HPLC-MS.

### Isolation and characterization of bioconversion products

~2 g of wet biomass of *Burkholderia* sp. MAK1 cells was resuspended in 100 ml of 10 mM potassium phosphate buffer, pH 7.2 supplemented with 15 mM of glucose and 0.25 mM of corresponding substrate and incubated at 30 °C. After bioconversion the cells of *Burkholderia* sp. MAK1 were separated by centrifugation. The supernatant liquid was vaporized to dryness under reduced pressure. The residue was dissolved in 5 ml of deionized water and purification of the product was carried out using reverse phase chromatography (12 g C-18 cartridge). Prior the purification the column was equilibrated with water. A mobile phase that consisted of water and methanol delivered in the gradient 10:0 → 10:5 elution mode. The collected fractions were analyzed by HPLC-MS. The fractions containing pure product were joined, and the solvent was removed under reduced pressure. ^1^H NMR spectra were recorded in DMSO-d_6_ or CDCl_3_ on Bruker Ascend 400, 400 MHz, and ^13^C NMR were recorded on Bruker Ascend 400, 100 MHz. Chemical shifts are reported in parts per million relative to the solvent resonance signal as an internal standard.

### HPLC-MS analysis

Before the analysis the cells were separated from the reaction mixture by centrifugation. The resultant supernatant was mixed with an equal part of acetonitrile, centrifuged and analyzed using a high performance liquid chromatography system (CBM-20A controller, two LC-2020AD pumps, SIL-30AC auto sampler and CTO-20AC column oven; Shimadzu, Japan) equipped with a photo diode array (PDA) detector (SPD-M20A Prominence diode array detector; Shimadzu, Japan) and a mass spectrometer (LCMS-2020, Shimadzu, Japan) equipped with an ESI source. The chromatographic separation was conducted using a YMC Pack Pro column, 3 × 150 mm (YMC, Japan) at 40 °C and a mobile phase that consisted of 0.1% formic acid water solution (solvent A), and acetonitrile (solvent B) delivered in the 0 → 60% gradient elution mode. Mass scans were measured from m/z 10 up to m/z 700, at 350 °C interface temperature, 250 °C DL temperature, ±4,500 V interface voltage, neutral DL/Qarray, using N_2_ as nebulizing and drying gas. Mass spectrometry data was acquired in both the positive and negative ionization mode. The data was analyzed using the LabSolutions LCMS software.

### Activity assay of pyridine-2,5-diol 5,6-dioxygenase from *Burkholderia* sp. MAK1

*Burkholderia* sp. MAK1 was grown at 30 °C for 20 hours in two 150 ml flasks, one containing 25 ml EFA medium (pyridin-2-ol induced cells), other containing 25 ml EFA medium where pyridin-2-ol is substituted for succinate (uninduced cells, negative control). The cells were harvested by centrifugation, washed twice with 10 mM potassium phosphate buffer (pH 7.2), suspended in 5 ml of the same buffer and sonicated. In 1.5 ml tubes three separate reaction mixtures were combined: internal control (990 μl 10 mM potassium phosphate buffer, pH 7.2 and 10 μl 2 mg/ml pyridine-2,5-diol solution), negative control (890 μl 10 mM potassium phosphate buffer, pH 7.2, 10 μl 2 mg/ml pyridine-2,5-diol solution and 100 μl cell-free extract of uninduced cells) and sample (890 μl 10 mM potassium phosphate buffer, pH 7.2, 10 μl 2 mg/ml pyridine-2,5-diol solution and 100 μl cell-free extract of induced cells). 100 μl of each reaction mixture was transferred to a 96 well plate and change in absorbance (λ_max_ 320 nm) per 30 minutes was measured. Overall change in absorbance was evaluated by subtracting noise data (internal and negative controls) from sample data. We were able to achieve 200–250 mU per 1 l medium, where 1 enzyme unit (U) is an amount of enzyme that catalyzes depletion of 1 μmol pyridine-2,5-diol per minute. The measured molar extinction coefficient of pyridine-2,5-diol in ethanol was 9800 M^−1^∙cm^−1^.

## Additional Information

**How to cite this article:** Stankevičiūtė, J. *et al*. Oxyfunctionalization of pyridine derivatives using whole cells of *Burkholderia* sp. MAK1. *Sci. Rep.* **6**, 39129; doi: 10.1038/srep39129 (2016).

**Publisher's note:** Springer Nature remains neutral with regard to jurisdictional claims in published maps and institutional affiliations.

## Supplementary Material

Supplementary Information

## Figures and Tables

**Table 1:** The bioconversion features of substituted pyridine-2-amines for which reactions products were detectable.

Substrate		Product			Conversion %
Name	[M + H]^+^	[M + H]^+^	NMR	Name/Possible outcome	
Pyridin-2-amine	95	111	−	Hydroxylated at the 5-position	99
3-Fluoropyridin-2-amine	113	129	−	Hydroxylated at the 5-position	43
3-Bromopyridin-2-amine	173 and 175	189 and 191	−	Hydroxylated at the 5-position	48
3-Chloropyridin-2-amine	129	145	+	6-amino-5-chloro-pyridin-3-ol	88
Pyridin-2,6-diamine	110	214	−	Hydroxylation followed by dimerization	88
4-Bromopyridin-2-amine	173 and 175	189 and 191	−	Hydroxylated at the 5-position	48
4-Fluoropyridin-2-amine	113	129	+	6-amino-4-fluoro-pyridin-3-ol	70
4-Chloropyridin-2-amine	129	145	+	6-amino-4-chloro-pyridin-3-ol	96
4-methyl-pyridin-2-amine	109	125	+	6-Amino-4-methyl-pyridin-3-ol	74
6-Bromopyridin-2-amine	173 and 175	189 and 191	−	Hydroxylated at the 5-position	53
6-Chloropyridin-2-amine	129	145	+	6-amino-2-chloropyridin-3-ol	89

For the NMR analysis approximately 10 mg of purified bioconversion product was used.

**Table 2:** The bioconversion features of substituted pyridine-2-ols for which reactions products were detectable.

Substrate		Product		Conversion %
Name	[M+H]^+^	[M+H]^+^	Possible outcome	
1-Methylpyridin-2-one	110	126	Hydroxylated at the 5-position	20
1-Ethylpyridin-2(1*H*)-one	124	140	Hydroxylated at the 5-position	17
1-Propylpyridin-2(1*H*)-one	138	154	Hydroxylated at the 5-position	Traces of product
3-(Trifluoromethyl) pyridin-2-ol	164	178 [M+H]^−^	Hydroxylated at the 5-position	46
3-amino-6-methyl-pyridin-2-ol	125	279	Hydroxylation followed by dimerization	88
Pyridine-2,6-diol	112	—	Hydroxylation followed by dimerization (blue pigment)	—

**Table 3:** The bioconversion features of pyridine and pyrazine derivatives for which reactions products were detectable.

Substrate		Product		
Name	[M+H]^+^	[M+H]^+^	NMR	Name/Possible outcome
Pyridine	80	96	−	Hydroxylated at the 1-position
2-Methylpyridine	94	110	−	Hydroxylated at the 1-position
3-Methylpyridine	94	110	−	Hydroxylated at the 1-position
4-Methylpyridine	94	110	−	Hydroxylated at the 1-position
Pyrazine	81	97 and 113	+	pyrazine-1-oxide and pyrazine-1,4-dioxide

For the NMR analysis approximately 10 mg of purified bioconversion product was used.
