# Supplementary material for: Oxyfunctionalization of pyridine derivatives using whole cells of Burkholderia sp. MAK1
Source: Sci Rep. 2016 Dec 16;6:39129. doi: 10.1038/srep39129 (PMC5159870; doi:10.1038/srep39129)
Supplement: Supplementary Information [file srep39129-s1.pdf]

Supplementary information for "Oxyfunctionalization of pyridine derivatives using whole cells of *Burkholderia* sp. MAK1"

Jonita Stankevičiūtė, Justas Vaitekūnas, Vytautas Petkevičius, Renata Gasparavičiūtė, Daiva Tauraitė, Rolandas Meškys

Department of Molecular Microbiology and Biotechnology, Institute of Biochemistry, the Life Sciences Centre, Vilnius University, Sauletekio al. 7, LT-10257 Vilnius, Lithuania

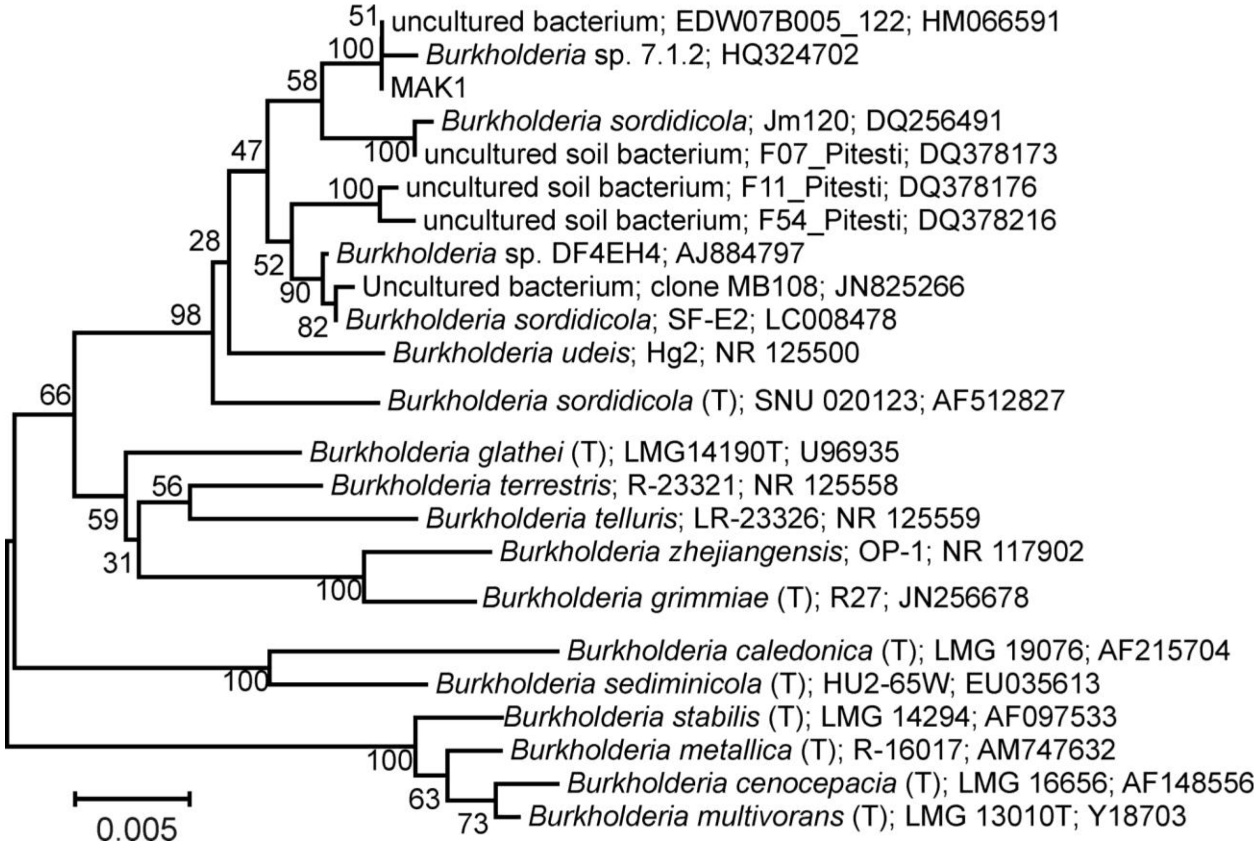

Figure S-I. Phylogenetic tree (constructed using the neighbour-joining method) showing the phylogenetic position of strain MAK1, based on 16S rRNA gene sequences.

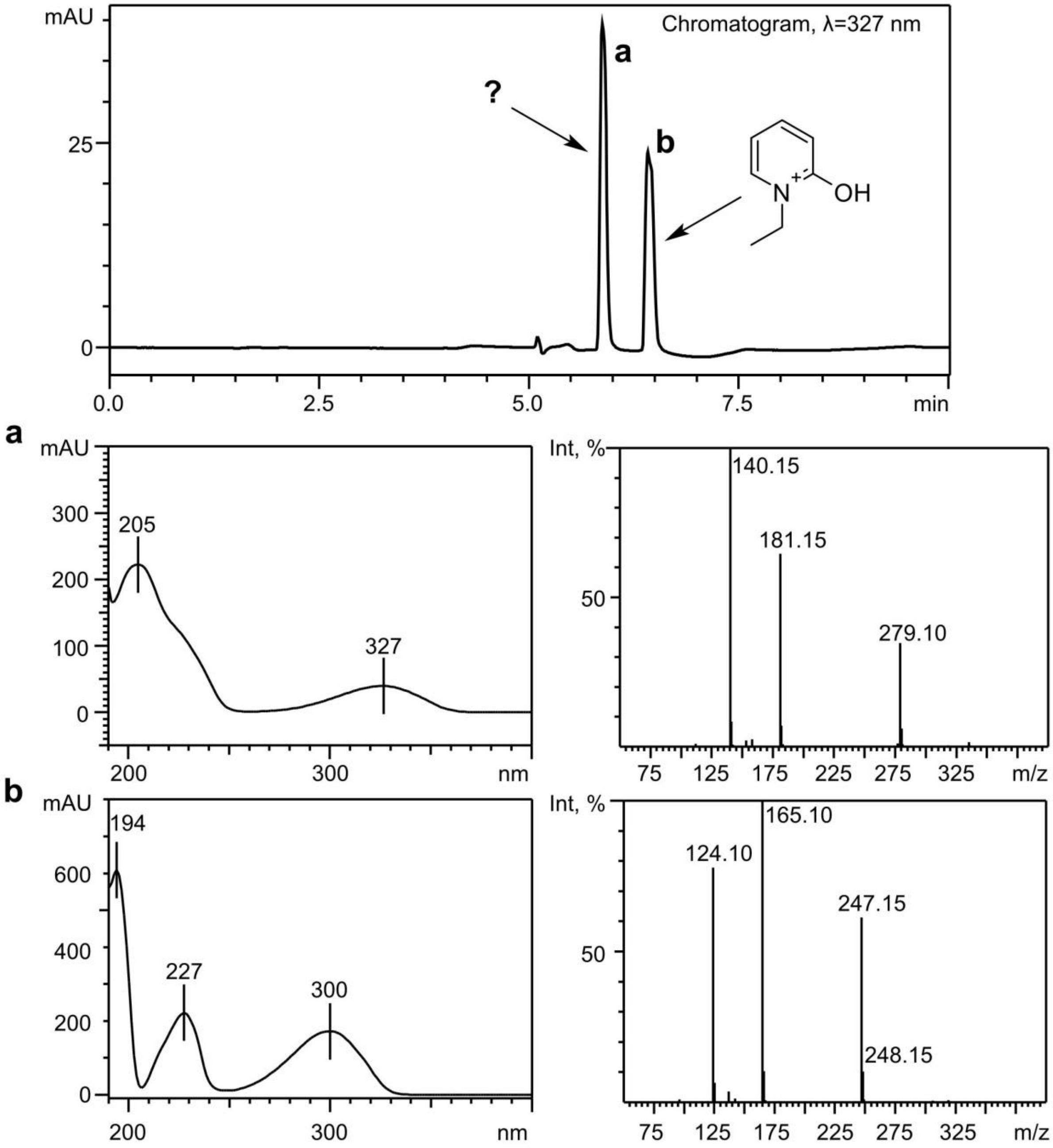

Figure S-II. Transformation of *N*-ethyl-2-hydroxypyridine by Burkholderia sp. MAK1. Analysis of the reaction mixture was performed by HPLC-MS. Chromatogram, UV-Vis spectra and mass spectra of (a) the product and (b) the substrate are presented.

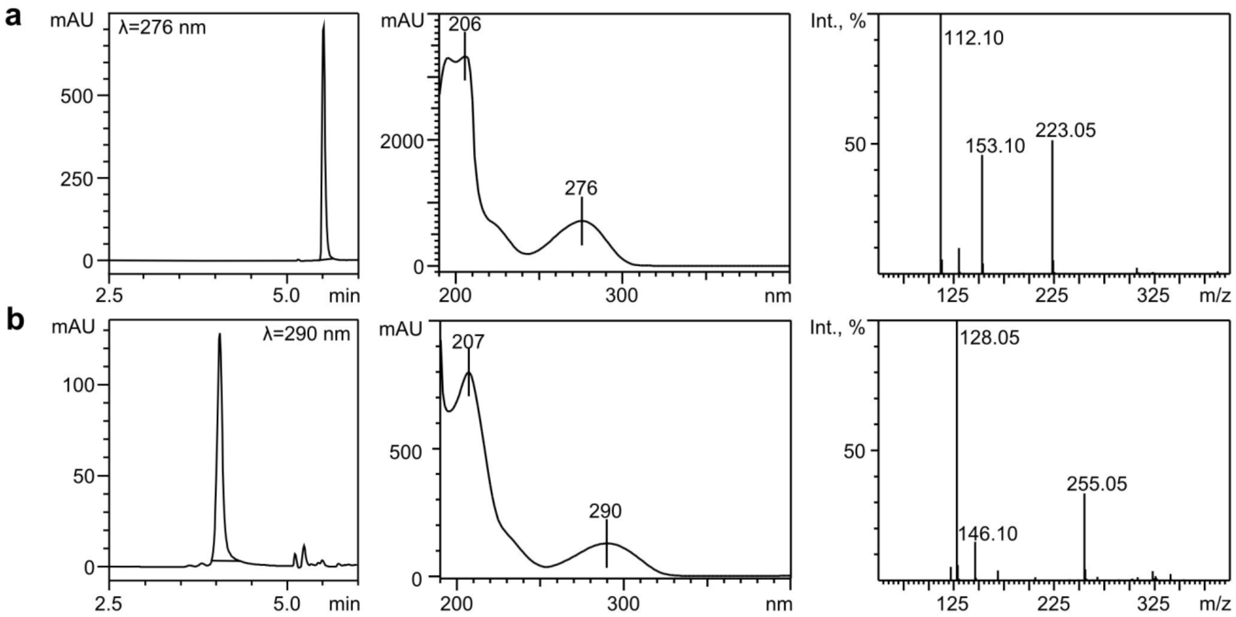

Figure S-III. Regioselective oxidation of 2,4-dihydroxypyridine by the cells of *Burkholderia* sp. MAK1. Analyses of the reaction mixture were performed by HPLC-MS (a) immediately after the addition of the substrate and (b) after 20 hours of incubation at 30 °C. Chromatograms and UV-Vis and mass spectra of the peaks are presented.

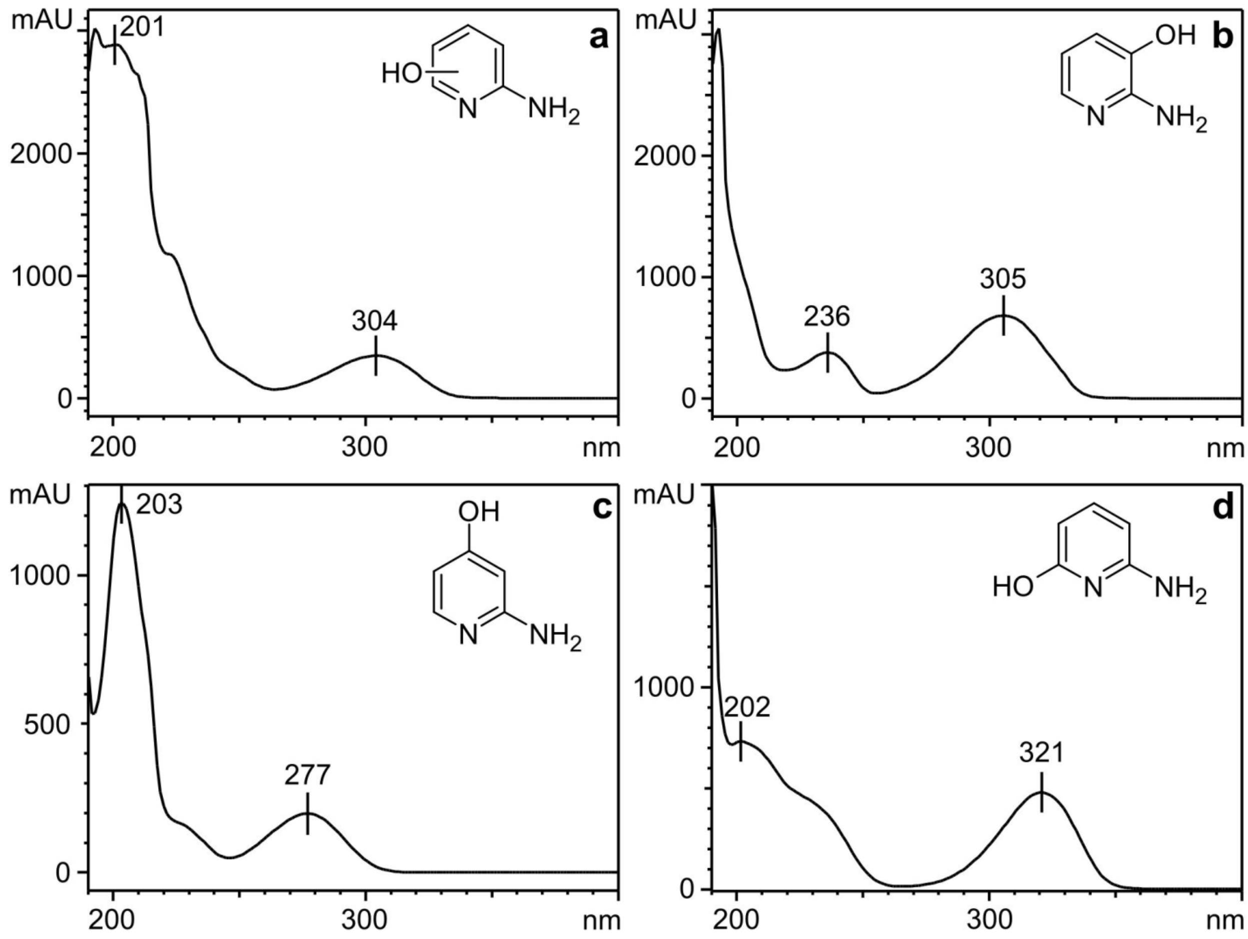

Figure S-IV. UV spectra of 2-aminohydroxypyridines. The UV spectrum of (a) 2-aminopyridine hydroxylated by *Burkholderia* sp. MAK1 cells and UV spectra of (b) 2-amino-3-hydroxypyridine, (c) 2-amino-4-hydroxypyridine, (d) 2-amino-6-hydroxypyridine standard solutions. The spectra were obtained by HPLC-MS. Note: the standard of 2-aminopyridin-5-ol is commercially unavailable.

Table S-1. Biochemical characterization of *Burkholderia* sp. MAK1.

According to API™ ZYM test (bioMerieux, USA), *Burkholderia* sp. MAK1 is alkaline phosphatase (2-naphthyl phosphate), esterase (C 4) (2-naphthyl butyrate), esterase (C 8) (2-naphthyl caprylate), leucine arylamidase (L-leucyl-2-naphthylamide), acid phosphatase (2-naphthyl phosphate), naphthol-AS-BI-phosphohydrolase (naphthol-AS-BI-phosphate), β-galactosidase (2-naphthyl-β-D-galactopyranoside), and *N*-acetyl-β-glucosaminidase (1-naphthyl-*N*-acetyl-β-D-glucosaminide) positive. *Burkholderia* sp. MAK1 cells show weak activities of lipase (C 14) (2-naphthyl myristate), valine arylamidase (L-valyl-2-naphthylamide), cystine arylamidase (L-cystyl-2-naphthylamide), α-galactosidase (6-Br-2-naphthyl-α-D-galactopyranoside), α-glucosidase (2-naphthyl-α-D-glucopyranoside), and α-fucosidase (2-naphthyl-α-L-fucopyranoside). The microorganism is negative for trypsin (*N*-benzoyl-DL-arginine-2-naphthylamide), α-chymotrypsin (*N*-glutaryl-phenylalanine-2-naphthylamide), β-glucuronidase (naphthol-AS-BI-β-D-glucuronide), β-glucosidase (6-Br-2-naphthyl-β-D-glucopyranoside), and α-mannosidase (6-Br-2-naphthyl-α-D-mannopyranoside).

According to API™ 50 CH test (bioMerieux, USA), *Burkholderia* sp. MAK1 is able to metabolize the following carbohydrates: D-arabinose, L-arabinose, D-xylose, D-galactose, D-glucose, D-fructose, D-mannose, L-rhamnose, dulcitol, inositol, D-mannitol, D-sorbitol, N-acetylglucosamine, amygdalin, esculin ferric citrate, salicin, D-cellobiose, D-maltose, D-lactose, D-melibiose, D-saccharose, D-trehalose, inulin, D-raffinose, amidon (starch), glycogen, xylitol, gentiobiose, D-turanose, D-lyxose, D-tagatose, D-fucose, D-arabitol, and potassium gluconate.

Burkholderia sp. MAK1 cells grow on D-glucose (0.1 %), D-galactose (0.1 %), *myo*-inositol (0.1 %), D-mannitol (0.1 %), D-mannose (0.1 %), D-sorbitol (0.1 %), D-fructose (0.1 %), D-xylose (0.1 %), succinate (0.1 %), and 2-hydroxypyridine (0.1 %) as sole carbon source but not on D-trehalose, D-ribose, 2-deoxy-D-glucose, D-melibiose, D-melezitose, sucrose, D-raffinose, D-maltose, D-cellobiose, and D-maltotriose.

Table S-2. The list of compounds which were tested as potential substrates for transformation by whole cells of *Burkholderia* sp. MAK1.

Substrate			Product
Name	Structure	$[M+H]^+$	$[M+H]^+$
Pyridin-2-ol	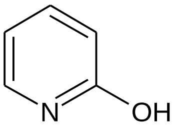	96	Substrate consumption occurs, no products detected
Pyridin-2-amine	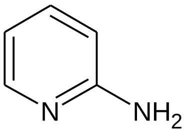	95	111
2-Methylpyridine	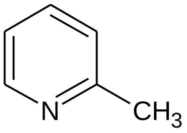	94	110
2-Chloropyridine	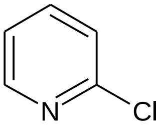	114	130
Pyridine-2-carboxylic acid	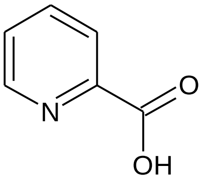	124	No reaction observed
2-Pyridylmethanol	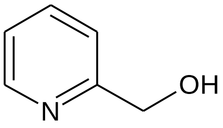	110	126<br>Slow transformation
2-Ethylpyridine	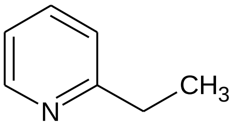	108	124

Pyridine-2-thiol	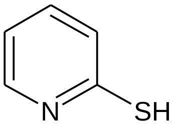	112	-
1-Methylpyridin-2-one	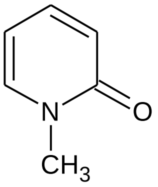	110	126
Pyridin-2-ol *N*-oxide	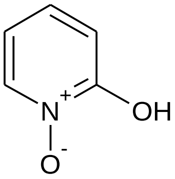	112	Substrate consumptio n occurs, no products detected
Pyridine-*N*-oxide	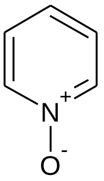	96	-
Pyridin-3-ol	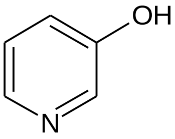	96	112
3-Bromopyridin-2-ol	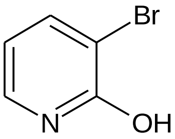	174 and 176	Substrate consumptio n occurs, no products detected
3-Methylpyridin-2-ol	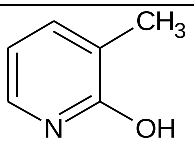	110	Substrate consumptio n occurs, no products detected
2-Hydroxypyridine-3-carboxylic acid	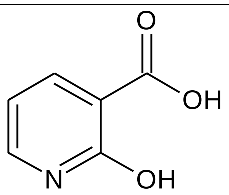	140	-

3-Methoxypyridin-2-ol	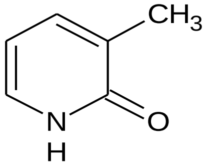	126	-
Pyridin-2,3-diol	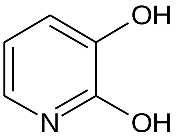	112	Substrate consumptio n occurs, no products detected
3-Methylpyridin-2-amine	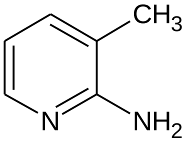	109	125
3-Methylpyridine-2-carbonitrile	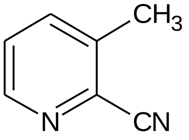	119	-
2,3-Dimethylpyridine	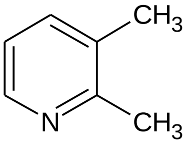	108	124<br>Traces of the product
2-Aminopyridin-3-ol	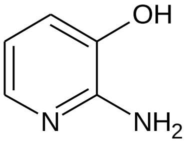	111	215
2-Aminopyridine-3-carbonitrile	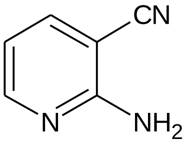	161 (as a cluster with acetonitrile)	-
Pyridin-2,3-diamine	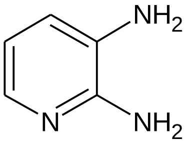	110	214
3-Nitropyridin-2-amine	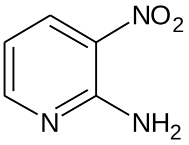	140	156

4-Methyl-3-nitro-pyridin-2-ol	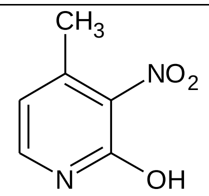	155	125
Pyridin-2,4-diol	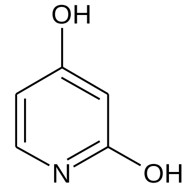	112	128
Pyridin-2,6-diol	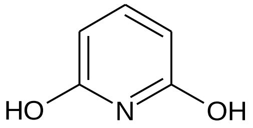	112	Substrate consumptio n occurs, blue color, no products detected
6-Methyl-pyridin-2-ol	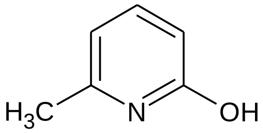	110	Substrate consumptio n occurs, no products detected
Pyrimidin-2-ol	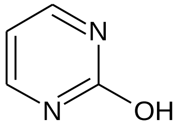	97	-
4-Methylpyridin-2-amine	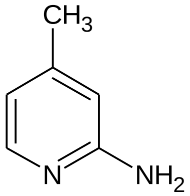	109	125
4,6-Dimethylpyridin-2-amine	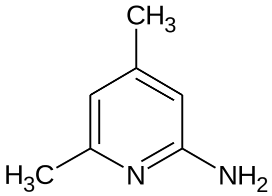	123	-
3-Methylpyridin-6-amine	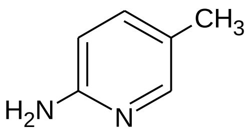	109	125<br>Slow transformati on

Pyridine	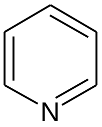	80	96
3-Methylpyridine	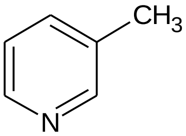	94	110
4-Methylpyridine	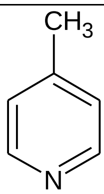	94	110
2,4-Dimethylpyridine	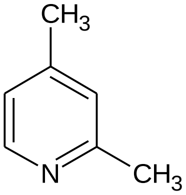	108	124
2,5-Dimethylpyridine	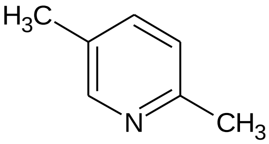	108	124
2,6-Dimethylpyridine	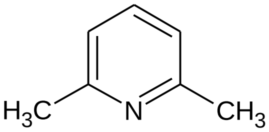	108	-
3,4-Dimethylpyridine	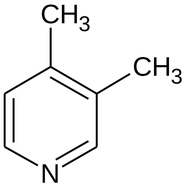	108	124
3,5-Dimethylpyridine	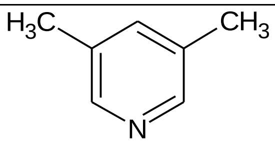	108	124
2,3,5-Trimethylpyridine	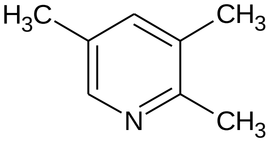	122	138<br>Traces of the product

2,4,6-Trimethylpyridine	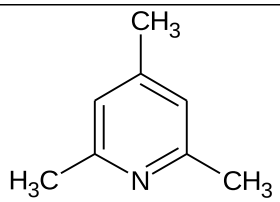	122	-
Pyran-2-one	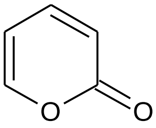	97	Substrate consumptio n occurs, no products detected
2-Hydroxy-6-oxo-1H-pyridine-4-carboxylic acid	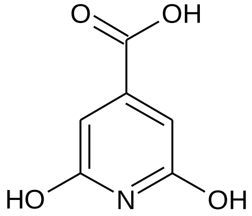	156	-
1-Propylpyridin-2(1*H*)-one	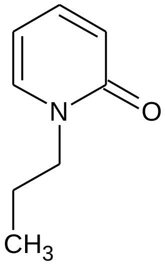	138	154<br>Traces of the product
6-Hydroxypyridine-2-carboxylic acid	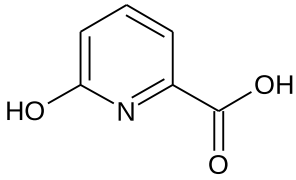	140	-
1-Butylpyridin-2(1*H*)-one	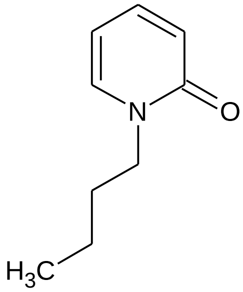	152	-

5-Hydroxypyridine-2-carboxylic acid	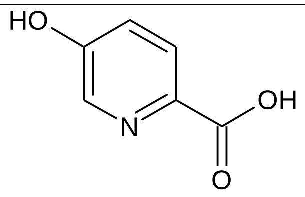	140	-
2-Hydroxy-6-methylpyridine-3-carbonitrile	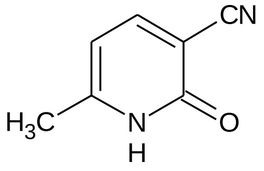	135	Substrate consumption is slow, no products detected
Pyridin-2,6-diamine	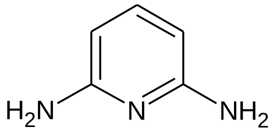	110	Substrate consumption occurs, no products detected
Pyrazine	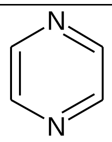	81	97;<br>113
1-Pyrazin-2-ylethanone	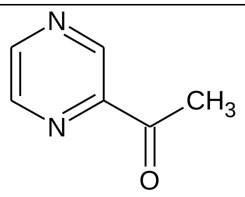	288	125
2,3-Dimethylpyrazine	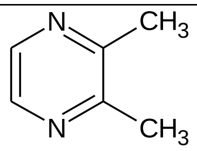	109	125<br>Traces of the product
2,5-Dimethylpyrazine	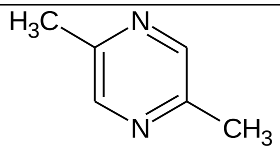	109	125<br>Traces of the product
2,6-Dimethylpyrazine	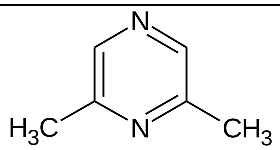	109	125

2,3,5-Trimethylpyrazine	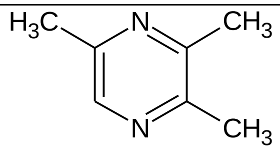	123	139<br>Traces of the product
Pyrazin-2-amine	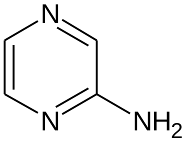	96	112
Tetramethylpyrazine	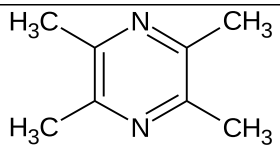	137	-
Pyridin-2-ol *N*-oxide	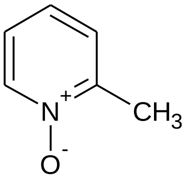	110	-
2-(2-Pyridyl)ethanol	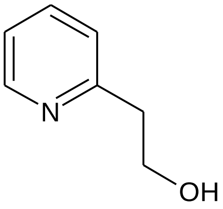	124	140<br>Slow transformation
3-Methylpyridazine	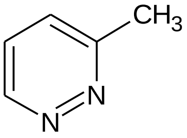	95	-
2-Methylpyridin-3-ol	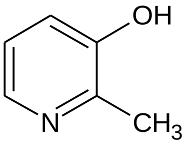	110	126
4-Methylpyridine-2-carbonitrile	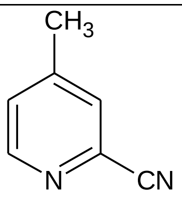	119	-

Pyridine-2,6-dicarbonitrile	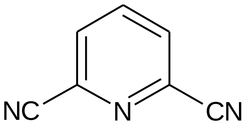	No substrate detected	-
4-Ethylpyridine	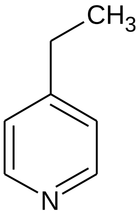	108	124
6,7-Dihydro-5H-cyclopenta[b]pyridine	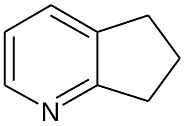	120	136
5-Methyl-6,7-dihydro-5H-cyclopenta[b]pyrazine	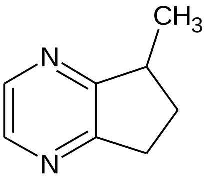	135	-
Pyrimidin-2,4-diamine	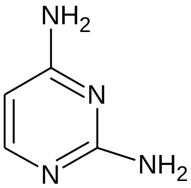	111	127
2-Methylpyridin-5-ol	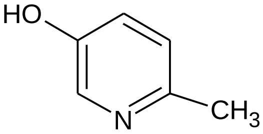	110	-
6-Hydroxypyridine-2-carboxylic acid	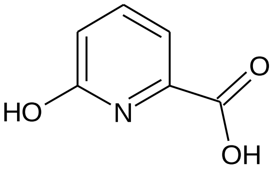	140	-
3-Hydroxypyridine-2-carboxylic acid	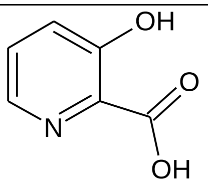	140	-

1-Ethylpyridin-2(1*H*)-one	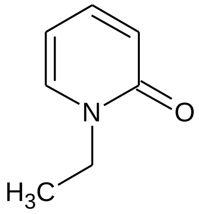	124	140
Pyridine-3-carboxamide	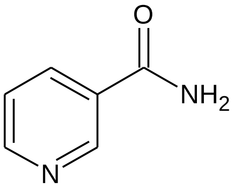	123	140
4-[(4-Nitrophenyl) methyl]pyridine	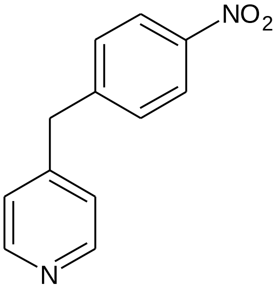	215	-
Pyridine-4-carboxylic acid	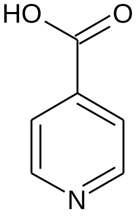	124	-
Pyridine-3-carbonitrile	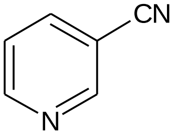	105	121
Pyridine-2,3-dicarboxylic acid	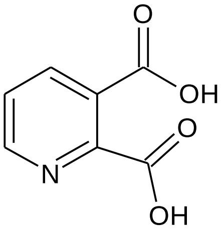	168	-
Pyridin-4-ol	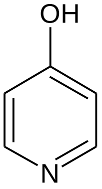	96	-

3-Fluoropyridin-2-ol	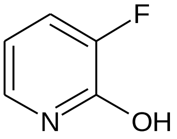	114	Substrate consumption occurs, no products detected
3-Chloropyridin-2-ol	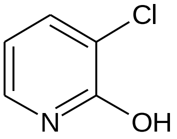	130	Substrate consumption occurs, no products detected
6-Chloropyridin-2-ol	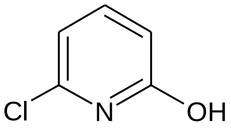	130	Substrate consumption occurs, green, no products detected
6-Bromopyridin-2-ol	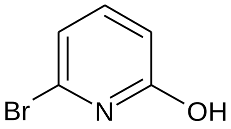	174 and 176	Substrate consumption occurs, green, no products detected
2-Aminopyridin-6-ol	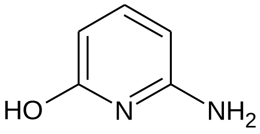	111	Substrate consumption occurs, several products formed
4-Chloropyridin-2-ol	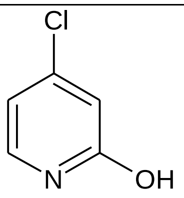	130	-
2-Oxo-1H-pyridine-4-carbonitrile	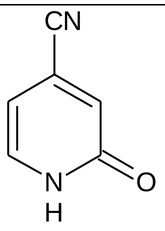	No substrate detected	-

4,6-Dimethylpyridin-2-ol	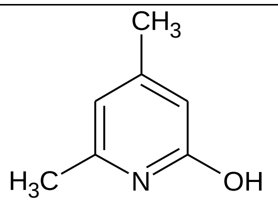	124	-
3-Amino-6-methyl-pyridin-2-ol	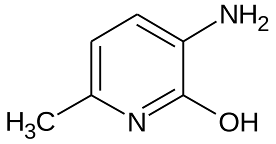	125	279
3-(Trifluoromethyl)<br>pyridin-2-ol	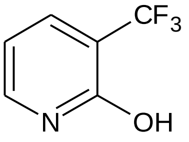	164	$[M-H]^-$:<br>178
4-(Trifluoromethyl) pyridin-2-ol	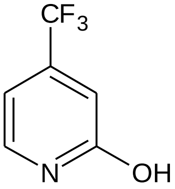	165	-
6-(Trifluoromethyl) pyridin-2-ol	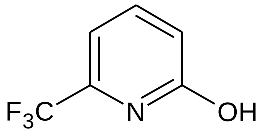	165	Substrate consumptio n occurs, no products detected
4-Chloropyridin-2-amine	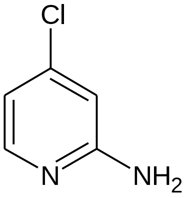	129	145
6-Bromopyridin-2-amine	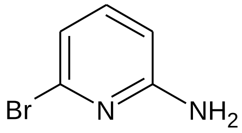	173 and 175	189 and 191
6-Chloropyridin-2-amine	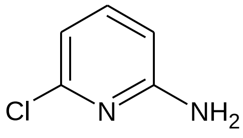	129	145

3-Bromopyridin-2-amine	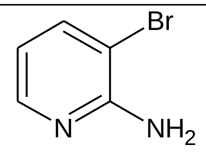	173 and 175	189 and 191
6-Fluoropyridin-2-amine	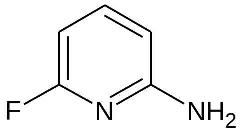	113	Substrate consumptio n occurs, no products detected
2-Aminopyridine-4-ol	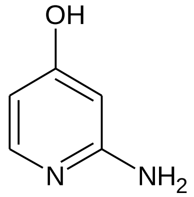	111	-
3-Chloropyridin-2-amine	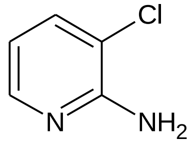	129	145
4-Fluoropyridin-2-amine	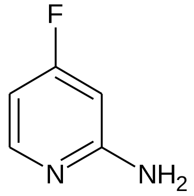	113	129
3,6-Dibromopyridin-2-amine	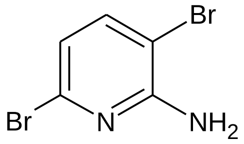	No substrate detected	?
3-Methoxypyridin-2-amine	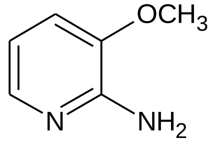	125	-
6-Methoxypyridin-2-amine	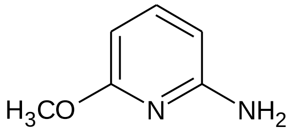	125	-
6-(Trifluoromethyl) pyridin-2-amine	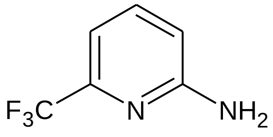	-	-

Ethyl 2-aminopyridine-3-carboxylate	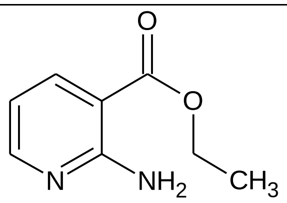	167	183<br><br>Traces of the product
2-Aminopyrimidin-4-ol	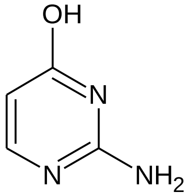	112	128
4-Chloropyrimidin-2-amine	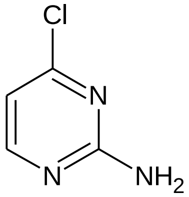	130	146<br><br>Traces of the product
4-Bromopyridin-2-amine	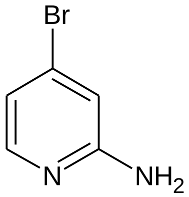	173 and 175	189 and 191
4-Bromopyridin-2-ol	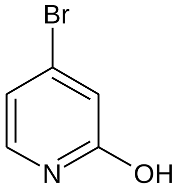	174 and 176	-
3-Fluoropyridin-2-amine	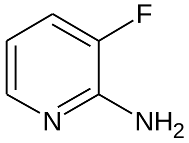	113	129<br><br>Two peaks with the same molecular mass
3-Bromo-6-methylpyridine-2-ol	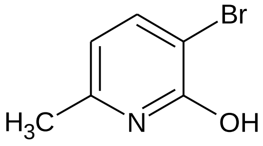	188 and 190	-

4-Methyl-3-nitro-pyridin-2-amine	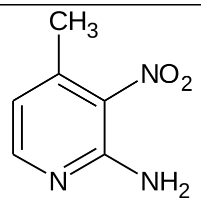	154	-

Table S-3. The efficiency of conversion of selected compounds by whole cells of *Burkholderia* sp. MAK1.

Name	Structure	Conversion, %
Pyridin-2-amine	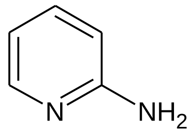	99
2-Pyridylmethanol	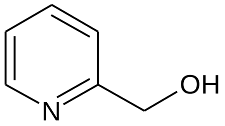	21
Pyridin-2,3-diol	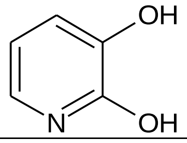	97
Pyridin-2,3-diamine	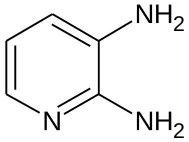	88
Pyridin-2,6-diamine	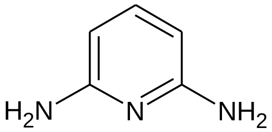	94
2,6-Dimethylpyrazine	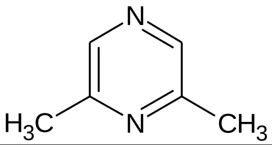	63
2-(2-Pyridyl)ethanol	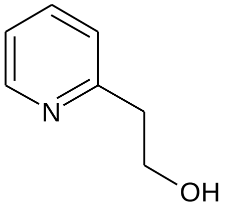	17
2-Methylpyridin-3-ol	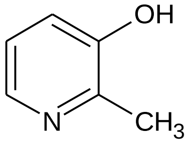	2
4-Ethylpyridine	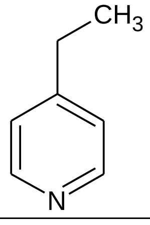	10
6,7-Dihydro-5H-cyclopenta[b]pyridine	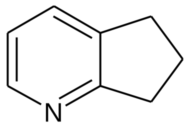	13

Pyrimidin-2,4-diamine	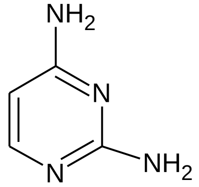	31
1-Ethylpyridin-2(1*H*)-one	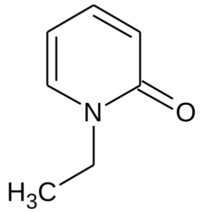	17
Pyridine-3-carbonitrile	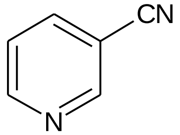	27
3-Amino-6-methyl-pyridin-2-ol	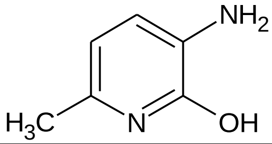	88
3-(Trifluoromethyl) pyridin-2-ol	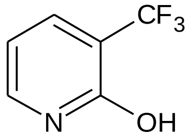	46
4-Chloropyridin-2-amine	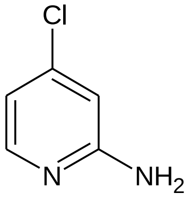	96
6-Bromopyridin-2-amine	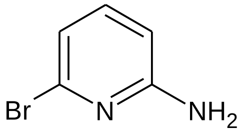	53
6-Chloropyridin-2-amine	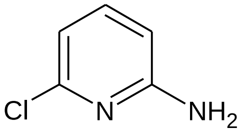	89
3-Bromopyridin-2-amine	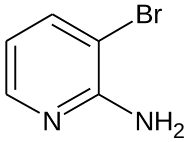	48
3-Chloropyridin-2-amine	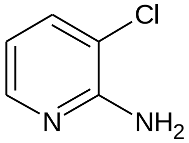	88

4-Fluoropyridin-2-amine	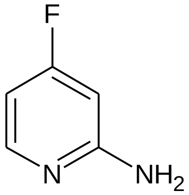	70
2-Aminopyrimidin-4-ol	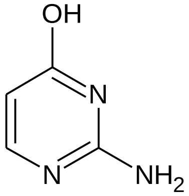	95
4-Bromopyridin-2-amine	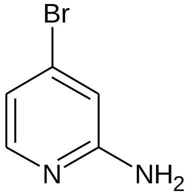	48
3-Fluoropyridin-2-amine	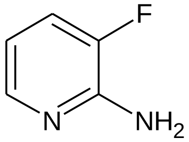	43
